# The Metabotropic Glutamate 5 Receptor Modulates Extinction and Reinstatement of Methamphetamine-Seeking in Mice

**DOI:** 10.1371/journal.pone.0068371

**Published:** 2013-07-04

**Authors:** Rose Chesworth, Robyn M. Brown, Jee Hyun Kim, Andrew J. Lawrence

**Affiliations:** 1 Behavioural Neuroscience Division, Florey Institute of Neuroscience and Mental Health, Parkville, Victoria, Australia; 2 Florey Department of Neuroscience and Mental Health, University of Melbourne, Parkville, Victoria, Australia; 3 Department of Neurosciences, Medical University of South Carolina, Charleston, South Carolina, United States of America; Tokyo Metropolitan Institute of Medical Science, Japan

## Abstract

Methamphetamine (METH) is a highly addictive psychostimulant with no therapeutics registered to assist addicts in discontinuing use. Glutamatergic dysfunction has been implicated in the development and maintenance of addiction. We sought to assess the involvement of the metabotropic glutamate 5 receptor (mGlu5) in behaviours relevant to METH addiction because this receptor has been implicated in the actions of other drugs of abuse, including alcohol, cocaine and opiates. mGlu5 knockout (KO) mice were tested in intravenous self-administration, conditioned place preference and locomotor sensitization. Self-administration of sucrose was used to assess the response of KO mice to a natural reward. Acquisition and maintenance of self-administration, as well as the motivation to self-administer METH was intact in mGlu5 KO mice. Importantly, mGlu5 KO mice required more extinction sessions to extinguish the operant response for METH, and exhibited an enhanced propensity to reinstate operant responding following exposure to drug-associated cues. This phenotype was not present when KO mice were tested in an equivalent paradigm assessing operant responding for sucrose. Development of conditioned place preference and locomotor sensitization were intact in KO mice; however, conditioned hyperactivity to the context previously paired with drug was elevated in KO mice. These data demonstrate a role for mGlu5 in the extinction and reinstatement of METH-seeking, and suggests a role for mGlu5 in regulating contextual salience.

## Introduction

Methamphetamine (METH) is a highly addictive psychostimulant for which there are currently no approved pharmacotherapies to treat abusers [1], [2]. Glutamatergic dysfunction has been implicated in the development and maintenance of addiction [3]. Indeed, overwhelming evidence from rodent models suggests chronic drug use results in the dysregulation of the glutamatergic system (e.g. [4]–[8]; for reviews see [3], [9]–[11]). This is reflected in human imaging studies, which reveal reduced brain glutamate concentrations in frontal white and grey matter in recently abstinent METH users [12]–[15]. Furthermore, relapse of drug-seeking in animal models can be attenuated by reversing glutamatergic dysfunction [16]–[18]. There is some support for this in preliminary human studies in drug addicts; N-acetylcysteine administration (which restores glutamate homeostasis) reduces cocaine craving in addicts [19], however, N- acetylcysteine combined with naltrexone for METH dependence has no effect on METH use or craving. Hence, while there may be a role for glutamate dysfunction in METH addiction (e.g. [12], [13], [20], [21]), the nature of this dysfunction requires further investigation.

Of the various ionotropic and metabotropic glutamate receptors, the metabotropic glutamate 5 receptor (mGlu5) has provoked considerable interest as a potential therapeutic target for drug addiction [22]–[28], partly due to its distribution in the neural circuitry underlying reward consumption and seeking. Specifically, mGlu5 is predominantly located post-synaptically [29], [30] in areas such as the hippocampus, cerebral cortex, nucleus accumbens (NAcc), lateral septum and the dorsal striatum [29], [31]. Moreover, mGlu5 has been implicated in drug-taking behaviour; a reduction in mGlu5 signalling reliably decreases drug-taking and drug-seeking behaviour for alcohol [32]–[34], cocaine [35], METH [36], [37], opiates [38] and nicotine [39], [40]. A reduction in the acquisition of conditioned place preference (CPP) for cocaine [41], [42] and morphine [43], as well as reduced expression of CPP for morphine [44], ethanol [45] and amphetamine [46] has also been reported following mGlu5 antagonist administration. Somatic signs of withdrawal to nicotine are attenuated [47], cocaine self-administration abolished [41] and ethanol consumption reduced [48] in mGlu5 KO mice.

Although those studies suggest that a *reduction* in mGlu5 signaling may be a helpful approach to treat drug abuse, it is important to highlight that mGlu5 receptors play a critical role in long-term potentiation and depression [49]–[53], the putative cellular mechanisms for learning and memory [54], [55]. Considering that addiction is characterised by dysfunction in learning processes [56], [57], the implication of mGlu5 in learning processes suggests mGlu5 signalling is a potential target for addiction therapeutics. In support of this idea, recent reports suggest a role for mGlu5 in extinction and reinstatement of drug-seeking behaviour. Administration of the mGlu5 positive allosteric modulator (PAM), 3-cyano-N-(1,3-diphenyl-1H-pyrazol-5-yl)benzamide (CDPPB), facilitates the acquisition and consolidation of extinction of cocaine self-administration [58], as well as enhancing extinction of cocaine CPP [59]. Unlike cocaine, CDPPB has no effect on extinction of METH self-administration [60]. Gass and colleagues [36] report reduced cue-induced and drug-primed reinstatement of METH self-administration following MTEP administration; yet extinction was not examined in that study.

The involvement of mGlu5 in behaviours relevant for METH addiction, particularly extinction and reinstatement, is not clear from the current pharmacological literature. In addition, issues of tolerance and dose have been raised with pharmacological approaches [60], [61]. Thus, in order to clarify the role of mGlu5 in these behaviours we utilised a model of genetic deletion. Specifically, the current study examined how mGlu5 KO mice responded to METH in a range of addiction-relevant behavioural paradigms. We also examined the response of mGlu5 KO mice to a natural reward (sucrose) in an operant paradigm, to delineate possible differences in extinction and reinstatement for METH and a natural reward. Using this genetic approach we sought to resolve if mGlu5 is necessary or sufficient for METH-driven behaviours.

## Methods

### Animals

mGlu5 KO mice [49] on a C57BL/6J background (Grm5tm1Rod; stock 003558) were obtained from Jackson Laboratories (Bar Harbour, ME, USA). All experimental subjects were fully backcrossed onto the C57BL/6 background (>10 generations). Mice were kept in standard housing (*ad libitum* standard laboratory chow and water, tissues for nesting material) under a 12∶12 h light-dark cycle unless otherwise specified. Experiments were conducted using age-matched adult male mice littermates; cohort 1 [WT (n = 15), mGlu5 KO (n = 22)] was used for conditioned place preference, cohort 2 [WT (n = 17), mGlu5 KO (n = 13)] was used for intravenous self administration, cohort 3 [WT (n = 8), mGlu5 KO (n = 7)] was used for sucrose self-administration. In all experiments, genotypes were counterbalanced across test apparatus and sessions, and were conducted by an experimenter blind to the genotype of the animals.

### Ethics Statement

All experiments were performed in accordance with the Prevention of Cruelty to Animals Act, 1986 under the guidelines of the National Health and Medical Research Council Code of Practice for the Care and Use of Animals for Experimental Purposes in Australia (Florey Animal Ethics Committee: ethics approval number: 11–015). All efforts were made to minimise animal suffering, to reduce the number of animals used, and to utilise alternatives to in vivo techniques, if available.

### Behavioural Phenotyping

#### Conditioned Place Preference (CPP)

The CPP apparatus (Lafayette Instruments, USA) consisted of two main compartments with differences in visual (wall patterns) and tactile (floor texture) cues, separated by a neutral compartment. The time spent in each compartment, as well as general locomotor activity, was recorded via horizontal optic sensor beams and specific software for the apparatus (Motor Monitor_TM_, Kinder Scientific, USA).

The CPP protocol was modified from that described previously [38], [62]. Before each session mice were habituated to the experimental room for at least 30 min. On day 1 (habituation), mice were placed in the central compartment and allowed free access to the entire apparatus. On days 2–4 (conditioning), mice received injections of saline (10 ml/kg) or METH (2 mg/kg i.p., dissolved in saline, Sigma-Aldrich Australia) and were immediately confined into one of the two conditioning compartments. A combination of unbiased and biased allocation was used. Specifically, mice with a neutral preference (45–55% for either side) were randomly allocated their drug-paired side (unbiased allocation). For the remainder of the mice, the drug was paired with the side which was least preferred (biased allocation); approximately 55% of mice demonstrated a side preference. On test, mice were given free access to the CPP apparatus.

All sessions were 30 min in duration and occurred at the same time each day. Place preference was calculated as a preference score (time spent in drug-paired zone- time spent in the saline-paired zone). Locomotor data was also collected throughout CPP testing to assess the development of behavioural sensitization [62].

#### Intravenous Self-Administration (IVSA)

Operant self-administration of oral sucrose or intravenous METH (3 µg/kg/infusion) was assessed using operant chambers (model ENV-307W, Med Associates, Vermont, USA) equipped with two levers, one paired with reinforcement (the active lever), the other resulted in no outcome when pressed (the inactive lever). A stimulus light located above the active lever was turned on for 10 s in conjunction with reinforcement (conditioned stimulus, CS). A vanilla-scented piece of paper (discriminative cue) was placed below the active lever prior to each session. The chambers were housed in sound attenuated boxes and ventilated with fans.

Self-administration procedures were conducted under a reverse dark-light cycle with singly housed mice, as published previously [38], [63]–[65]. Mice were given at least 7 days to acclimatise to the reverse light cycle and to single-housing. All sessions were conducted during the first half of the dark cycle. Mice were taught to discriminate the active from inactive lever with 8 days of sucrose training [38], [63]–[65], to ensure differences in METH self-administration were not due to an inability to learn an operant task. The volume of sucrose delivered was 5 µl, over 1.7 s. Inclusion criteria were75% discrimination for the active lever vs. the inactive lever with >100 active lever presses per day, for the last 3 days of training. Sucrose training sessions were 2 h.

After instrumental training, mice were anaesthetised using isoflurane (1.5–2.0% in air) plus meloxicam (3 mg/kg i.p.) and then implanted with indwelling venous cannulae as previously described [38], [63]–[65]. Mice were treated with neomycin antibiotic diluted in saline following surgery and during the 4 days recovery post-surgery, prior to the commencement of behavioural experiments.

For self-administration testing, mice were connected via the jugular catheter to an intravenous line (Tygon; Saint Gobain Performance Plastics, Campbellfield, VIC, Australia) which in turn was connected to a 22 gauge swivel (Instech Solomon, Plymouth Meeting, PA, USA). The swivel was connected with BCOEX-T22 tubing to a syringe filled with methamphetamine solution held in an infusion pump. Following recovery from surgery, mice were tested using an FR1 schedule of reinforcement. Infusion volume was 19 µl and duration of infusion 1.7 s. Sessions were terminated if a predetermined maximum number of drug infusions (100) was attained, and no drug was administered in the 10 s immediately after each drug infusion. During this period the stimulus light remained active, and any active lever presses were recorded as ‘time out’ responses. All sessions were two hours in length (maximum infusion contingency notwithstanding). Mice were given a maximum of 12 days to reach the following criteria and be considered as having ‘acquired’ the lever press response for METH: >6 infusions, with 75% discrimination for the active lever, maintained over three consecutive days. Mice that did not reach criteria were excluded from the study. Data collected from the three days during which mice met criteria were considered ‘FR1 Acquisition’. Mice were then tested for 5 days under a fixed ratio 1 (FR1) schedule to assess ‘Stable FR1’ responding. This was followed by two days of progressive ratio (PR) responding, interspersed with one day of FR1, to assess the motivation to self-administer METH (see [38], [63]–[65] for methods). Breakpoint was used to assess motivation to self-administer, and was defined as the point where an animal ceases to press the active lever for a drug infusion when the instrumental requirement is progressively increased [38], [63]–[65]. Extinction training followed PR testing, where responses on the active lever were no longer reinforced with a drug infusion. The stimulus light and discriminative cue were not present during extinction sessions. Extinction sessions ran for 45 min. Mice needed to reach extinction criteria to be considered extinguished: 30% of averaged Stable FR1 active lever presses maintained over two consecutive days [66]. The day after extinction criteria was met, reinstatement testing (one hour) was conducted, where the stimulus light and vanilla discriminative stimulus were reintroduced to the operant chambers, but active lever responses remained unreinforced. Mice were considered to have reinstated their operant responding if their active lever presses during the reinstatement test were double the number of active lever responses during extinction, and at least ten active lever presses were made [67]. Throughout the experiment mice were tested periodically for patency using 0.02–0.03 ml of 15 mg/kg ketamine (Parnell Laboratories, Alexandria, Australia); if signs of hypnosis were not apparent mice were excluded from the study.

A third cohort of mice was tested for self-administration of 10% sucrose w/v. Experimental procedures were identical to IVSA procedures; however, mice did not undergo jugular catheter surgery. Also, the maximum number of sucrose deliveries was increased to 550 during FR1 acquisition and Stable FR1 training.

### Statistical Analysis

Three- and two-way repeated measures (RM) analysis of variance (ANOVA) with factors ‘days’, ‘lever type’ and/or ‘drug’ and between factor ‘genotype’ were conducted. Where appropriate, this was followed by one-way ANOVA split by corresponding factor with a Bonferroni correction (*p* = .05/number of independent variables). One-way ANOVA with the between factor ‘genotype’ was used to assess latency to acquisition/extinction. A log-rank (Mantel-Cox) test was used to assess duration of extinction, and Fisher’s exact test was used assess the propensity to reinstate. Data presented as means±standard error of the mean (SEM). Data analysis conducted using SPSS Statistics version 20 and GraphPad: Prism version 5.

## Results

### CPP

**Figure 1:**
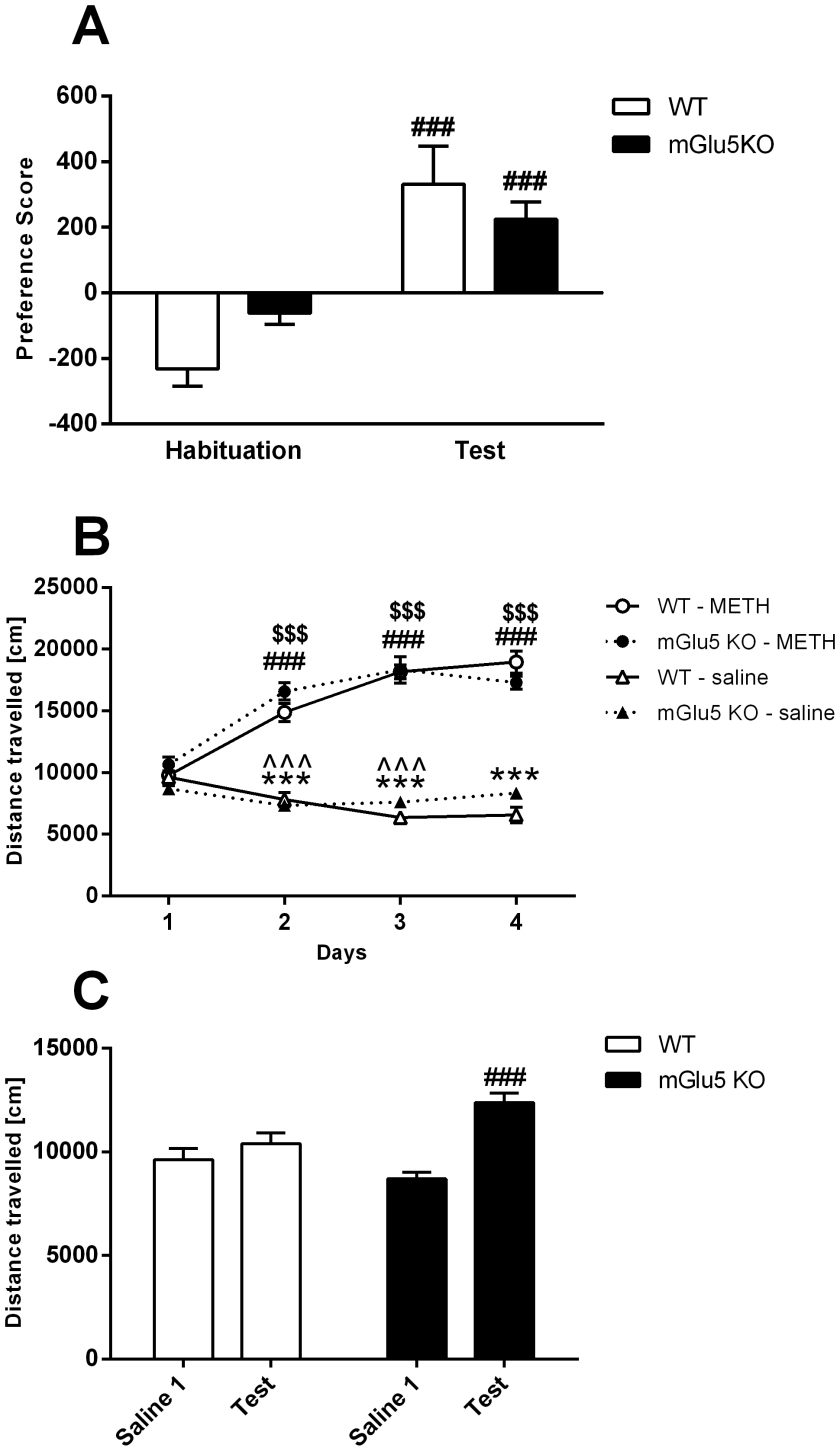
METH CPP Preference Score, Locomotor Sensitization and Conditioned Hyperactivity. **A)** Preference score in WT and mGlu5 KO mice at habituation and test. Preference score is defined as (time spent in the METH-paired compartment – time spent in the saline-paired compartment). **B)** Locomotor sensitization in WT and mGlu5 KO mice following acute saline or 2 mg/kg METH i.p. injection over 4 consecutive days. **C)** Conditioned hyperactivity in WT and mGlu5 KO mice during the CPP test. Data (mean±SEM) analysed using two- or three-way RM ANOVA, followed by one-way ANOVA split by the factor ‘genotype’ with a Bonferroni correction. In Figs **A)** and **C)**, significant effects of ‘day’ (*vs.* habituation) are represented by “#” (^###^ *p*<.001); significant effects of ‘genotype’ (*vs*. WT on the same day) are represented by ‘*’ (**p<.01). In Fig **B)**, significant effects of ‘day’ (vs. METH day 1) are indicated by ‘$’ for WT mice (^$$$^ *p*<.001), ‘#’ for mGlu5 KO mice (^###^ *p*<.001). Significant effects of ‘day’ (vs. saline day 1) are indicated by ‘*’ for WT mice (***p<.001) and ‘?’ for mGlu5 KO mice (???*p*<.001). A significant ‘day’ × ‘genotype’ interaction was present in both Fig. A) and C), suggesting **A)** a greater change degree of change from habituation to test in WT compared to mGlu5 KO mice, and **C)** a greater change degree of change from habituation to test in mGlu5 KO compared to WT mice. Abbreviations: Saline 1 - day 1 of saline treatment.

Preference for the METH-paired compartment during the CPP test was significantly increased from preference during habituation in both genotypes, evidenced by a main effect of ‘day’ [F(1,35) = 59.2, *p<*.001] and no effect of ‘genotype’ [F(1,35) = .1, *p = *.8]. A significant interaction [F(1,35) = 7.1, *p = *.01] suggests a greater increase in preference score in WT compared to mGlu5 KO mice; however, one-way ANOVA split by ‘genotype’ demonstrates a significant increase in preference score in both genotypes [WT: F(1,14) = 28.5, *p<*.001; mGlu5 KO: F(1,21) = 25.6, *p<*.001; Fig. 1a].

### Locomotor Sensitization

Hyperactivity in mGlu5 KO mice was present upon exposure to a novel environment [‘time’ × ‘genotype’ interaction, F(1,175) = 6.1, *p = *.001; data not shown], similar to the phenotype of this mouse reported previously [68]. The development of sensitization to METH (2 mg/kg i.p.) was present in both genotypes [main effect of ‘days’ F(3,105) = 26.4, *p<*.001, no effect of ‘genotype’ F(1,35) = .7, *p = *.4; Fig. 1b]. Locomotor activity was heightened following METH administration compared to saline administration [main effect of ‘drug’ F(1,35) = 479.1, *p<*.001]. A significant interaction between ‘drug’ × ‘days’ [F(3,105) = 106.6, *p<*.001] suggests locomotor activity increased under METH compared to saline treatment during conditioning (Fig. 1b). Indeed, one-way ANOVA split by ‘drug’ and ‘genotype’ revealed a significant increase in locomotor activity on METH conditioning days (*vs*. METH conditioning day 1), and a decrease in locomotor activity on saline conditioning days (*vs*. saline conditioning day 1) in both genotypes (Fig. 1b).

### Conditioned Hyperactivity

Conditioned hyperactivity was present on CPP test day [main effect of ‘day’ F(1,35) = 48.4, *p<*.001; not of ‘genotype’ F(1,35) = .9, *p = *.3; Fig 1c]. There was a significant ‘day’ × ‘genotype’ interaction [F(1,35) = 20.8, *p<*.001], suggesting conditioned hyperactivity on test day was more pronounced in mGlu5 KO mice (Fig. 1c). A significant ‘day’ × ‘time’ × ‘genotype’ interaction [F(5,175) = 2.4, p = .04] suggests conditioned hyperactivity was present in KO mice throughout the entire test session, but was only present in WT mice in the first 5 minutes of the test (data not shown).

### Instrumental Learning

**Figure 2:**
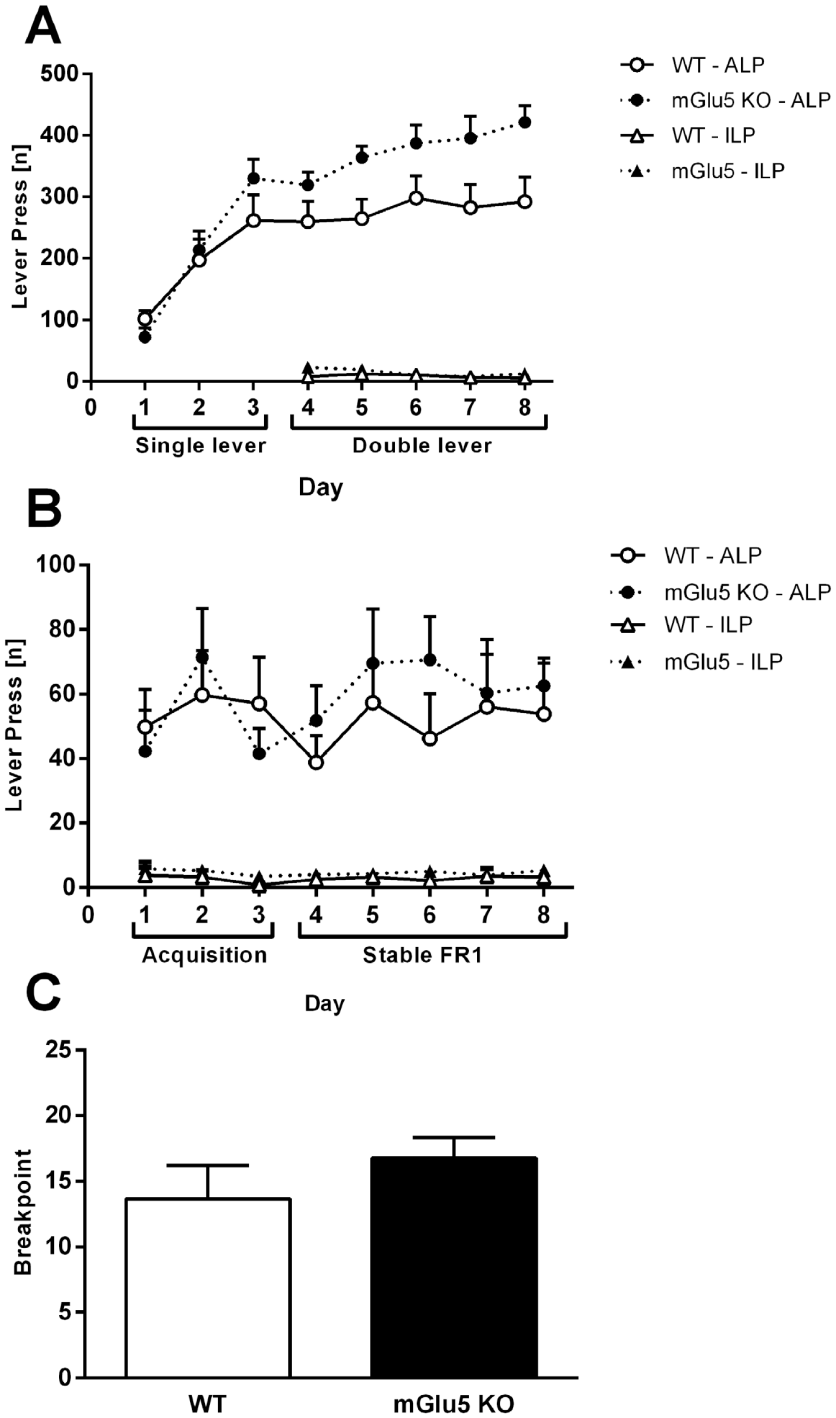
Instrumental learning, METH self-administration and progressive ratio testing. **A)** Self-administration of 10% sucrose solution with a fixed ratio 1 schedule in WT and mGlu5 KO mice. Single lever training (days 1–3) was followed by double lever training (days 4–8). A significant main effect of ‘lever type’ suggests clear discrimination for the active lever in each genotype**. B)** Acquisition and stable self-administration of 3 µg/kg/infusion METH in WT and mGlu5 KO mice. **C)** Final breakpoint reached within a two-hour test using a progressive ratio schedule of reinforcement. Data (mean±SEM) analysed using two- or three-way RM ANOVA, followed by one-way ANOVA split by the factor ‘genotype’ with a Bonferroni correction where appropriate. Significant main effects of ‘lever type’ during sucrose and METH acquisition and self-administration suggest clear discrimination for the active lever in each genotype.

Acquisition of the single lever instrumental response was similar between the genotypes [main effect of ‘days’ F(2,58) = 50.6, p<.0001, n.s. main effect of ‘genotype’ F(1,28) = .2, p = .7; Fig. 2a]. During double lever training, both genotypes showed clear discrimination for the active lever over the inactive lever [main effect of ‘lever type’ F(1,28) = 295.7, *p<*.001; n.s. main effect of ‘days’ F(4,116) = 1.9, p = .1, Fig. 2a]. mGlu5 KO mice made more active lever presses than WT mice on days 4–8 [main effect of ‘genotype’ F(1,28) = 6.7, p = .02; interaction between ‘genotype’ and ‘lever type’ F(1,28) = 5.3, p = .03; Fig. 2a].

*IVSA.* 3 WT mice were excluded as they did not reach FR1 acquisition criteria; data from these mice was excluded from instrumental analyses. 3 mice were excluded due to loss of patency during the experiment; data for these mice was included in analyses until mice lost patency. 1 WT was excluded from the extinction analysis, as its score was >14 standard deviations above the mean. The data from this mouse was also excluded from the reinstatement analysis.

During FR1 acquisition, there was no difference in the number of lever presses made or infusions received between the genotypes [no main effect of ‘genotype’ for lever press: F(1,22) = 0.1, *p = *.9; infusions: F(1,22) = 1.1, *p = *.3; Table 1]. Both genotypes showed clear discrimination for the active lever over the inactive lever [main effect of ‘lever type’ F(1,22) = 73.0, *p<*.001; Fig 2b]. During Stable FR1 training, both genotypes made a similar number of infusions [WT: 42.2±6.5; mGlu5 KO: 43.8±6.1; *p>*.05] and showed clear discrimination for the active lever over the inactive lever [main effect of ‘lever type’, F(1,20) = 63.4, *p<*.001; Fig. 2b]. There was no difference between the genotypes in the motivation to self-administer METH, as assessed by a PR [n.s. main effect of ‘genotype’ F(1,18) = .1, p = .7; n.s. effect of ‘days’ F(1,18) = .8, p = .4; data presented as average breakpoint across two days of PR; Fig. 2c].

**Table 1:** Lever Responses and Infusions during METH IVSA FR1 Acquisition, Self-administration, Extinction and Reinstatement.

Measure	WT	mGlu5 KO
FR1 Acquisition Infusions	40.5±6.1	32.8±4.1
Stable FR1 Infusions	39.9±6.2	43.8±6.1
Stable FR1 ALP per minute	.48+.13	.52+.07
Extinction ALP per minute	.11+.04 $	.16+.03 $$
Extinction Final ALP	4.6±1.5	6.9±1.5 ##
Extinction Final ILP	1.3±1.0	2.0±0.6
Reinstatement ALP	118.5±94.5	23.8±3.4 ###
Reinstatement ILP	4.0±4.0	3.2±1.6

Stable FR1 infusions averaged over 5 days of stable self-administration; extinction data averaged over the final two days of extinction. Data presented as means±SEM. Data analysed using two- or three-way RM ANOVA, followed by one-way ANOVA split by corresponding factor, where appropriate. Significant effects of ‘lever type’ are indicated by hash symbols (*vs*. inactive lever; ^##^ *p*<.01, ^###^ *p*<.001); significant effects of ‘genotype’ are indicated by asterisks (*p<.05); significant effects of ‘day’ are indicated by ‘$’ (^$^ *p*<.05). Abbreviations: ALP - active lever press; FR1 - fixed ratio 1; ILP - inactive lever press.

**Figure 3:**
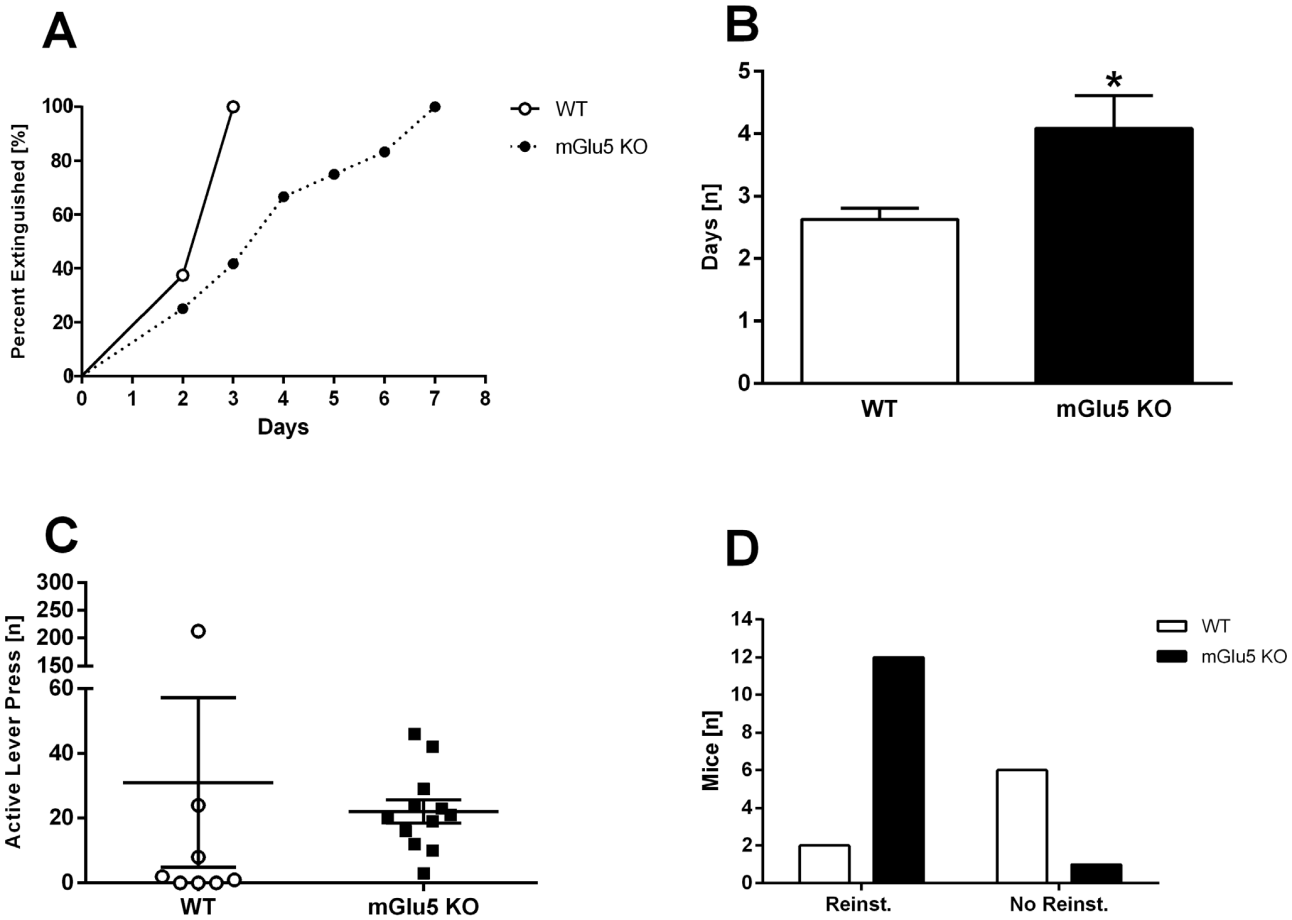
Extinction and reinstatement of the operant response for METH. **A)** Percentage of mice extinguished per day. mGlu5 KO mice took a significantly greater number of days to extinguish the operant response. Data (mean±SEM) analysed using a log-rank test; there was a significant effect of ‘genotype’ (*p* = .03). **B)** Average number of days to extinguish the operant response. mGlu5 KO mice took significantly longer to extinguish their operant responding than WTs. Data (mean±SEM) analysed using one-way ANOVA; a significant effect of ‘genotype’ indicated by ‘*’ (*p<.05). **C)** Active lever pressing during reinstatement. Data (mean±SEM) analysed using one-way ANOVA. **D)** Proportion of WT and mGlu5 KO mice meeting reinstatement criteria. Data analysed using a Fisher’s exact test. Abbreviations: Reinst - mice which met reinstatement criteria; No Reinst - mice which did not meet reinstatement criteria.

During extinction training, mGlu5 KO mice demonstrated a significantly longer latency to extinguish their responding for drug reinforcement [log-rank test, χ^2^ = 5.0, df = 1, *p = *.03; Fig. 3a]. This was confirmed with a one-way ANOVA main effect of ‘genotype’ on the latency to extinguish [F(1,18) = 4.7, *p = *.04; Fig. 3b]. Lever presses on the final two days of extinction training were averaged to produce an extinction lever press score. In order to compare Stable FR1 responding to extinction, responding was expressed as lever presses per minute to account for session duration. Both genotypes demonstrated a significant reduction in their extinction lever press score compared to averaged Stable FR1 [main effect of ‘day’ F(1,18) = 41.0, *p*<.001; no main effect of ‘genotype’ F(1,18) = .3, *p = *.6; Table 1]. During reinstatement, where cues signalling the availability of drug reinforcement were reintroduced, there was a low level of lever pressing in WT mice (Fig. 3c; n.s. effect of ‘genotype’). We applied reinstatement criteria (at least 2x extinction score and >10 active lever presses) to assess differences in the propensity to reinstate. The number of mGlu5 KO that met reinstatement criteria was greater than WT mice (2/8 WT mice and 12/13 mGlu5 KO mice reinstated; Fisher’s exact test: *p* = .004; Phi coefficient: −0.685; Fig. 3d). Examining mice that did meet reinstatement criteria, both genotypes showed enhanced responding on the active lever during reinstatement compared to that of the final two days of extinction training [main effect of ‘day’ F(1,11) = 16.0, *p* = .002]. There was a main effect of ‘genotype’ [F(1,11) = 8.7, *p* = .01], and a ‘genotype’ × ‘day’ interaction [F(1,11) = 8.5, *p* = .01], suggesting lower active lever pressing during reinstatement in mGlu5 KO mice compared to WT upon reinstatement (Table 1).

### Sucrose Self-administration

Both genotypes acquired the operant response for 10% sucrose solution during the initial 8 days of training, with clear discrimination for the active lever (data not shown). During FR1 acquisition and Stable FR1, the number of sucrose deliveries self-administered was similar between the genotypes [FR1 acquisition: WT: 241±42, mGlu5 KO: 228±21, F(1,13) = .1, *p* = .8; Stable FR1: WT: 271±36, mGlu5 KO: 215±31, F(1,13) = 1.3, *p* = .3]. There was clear discrimination for the active lever in both genotypes during Stable FR1 [main effect of ‘lever type’ F(1,13) = 68.5, *p*<.0001; no interaction; data not shown]. The motivation to self-administer sucrose demonstrated no difference in the breakpoint between the two genotypes [average breakpoint WT: 34.8±3.4, mGlu5 KO: 28.2±2.5, unpaired t-test, t = 1.5, df = 13, *p* = 2].

**Figure 4:**
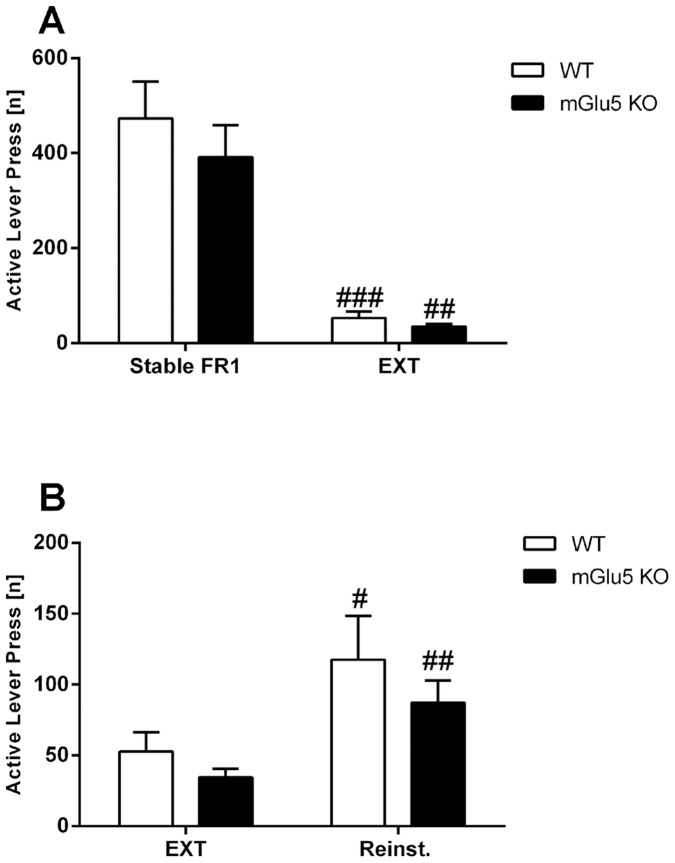
Extinction and reinstatement of the operant response for sucrose. **A)** Average active lever presses during Stable FR1 and the final 2 days of extinction. Both genotypes significantly decreased their active lever presses during both days of extinction. **B)** Average active lever presses during extinction and cue-induced reinstatement. Both genotypes increased their active lever pressing in the reinstatement test following extinction. Data (mean±SEM) analysed using two-way RM ANOVA, followed by one-way ANOVA split by the factor ‘genotype’ with a Bonferroni correction. Significant effects of ‘day’ (*vs.* Stable FR1) are represented by “#” (^##^ *p*<.01; ^###^ *p*<.001). Abbreviations: EXT - final extinction score; FR1– fixed ratio 1; Reinst - reinstatement.

Unlike METH IVSA, both genotypes met extinction criteria within two days of testing (percentage of Stable FR1 day 1 WT: 15.9±4.3, mGlu5 KO: 9.5±1.9, day 2: WT: 16.1±4.2, mGlu5 KO: 11.4±3.1). The extinction lever press score was significantly reduced from that of Stable FR1 in both genotypes [main effect of ‘day’ F(1,13) = 67.5, *p*<.0001 but not of ‘genotype’ F(1,13) = .7, *p* = .4, no interaction; Fig. 4a].

In the reinstatement test, there was a similar proportion of WT and mGlu5 KO mice which met reinstatement criteria (WT: 5/8, mGlu5 KO: 6/7; Fisher’s exact test *p* = .6; Phi coefficient: −0.134). Within the reinstating mice, both genotypes demonstrated significantly greater active lever pressing during reinstatement compared to extinction [main effect of ‘day’ F(1,10) = 39.5, *p*<.001; Fig. 4b]. This response was not different between the genotypes [no main effect of ‘genotype’ F(1,10) = .9, p = .4; no interaction; Fig. 4b].

## Discussion

The current study provides evidence for a distinct role for mGlu5 in the extinction of operant responding for METH, but not sucrose. mGlu5 KO mice showed an enhanced propensity for cue-induced operant METH- but not sucrose-seeking. KO mice also demonstrated enhanced conditioned hyperactivity to a previously METH-paired context. Interestingly, mGlu5 does not appear critical for the self-administration or motivation to self-administer METH. Furthermore, loss of mGlu5 signalling does not impair the acquisition of CPP or development of locomotor sensitization to METH. The phenotype observed suggests impaired inhibition of METH-seeking operant behaviour in the absence of METH. Additionally, mGlu5 KO mice show augmented or enduring responding to METH-paired cues and contexts in the absence of the drug, putatively suggesting a role for mGlu5 in mediating the salience of environmental cues and contexts associated with drug availability.

### mGlu5 is not Critical for Acquisition of Operant Responding, Place Preference or Locomotor Sensitization

mGlu5 KO mice did not demonstrate altered acquisition or motivation to self-administer METH, nor was the acquisition of METH CPP, development of locomotor sensitization or expression of sensitization different to WT mice. This suggests mGlu5 may be sufficient, but not necessary, for the development of these METH-induced behaviours, as acute pharmacological studies have demonstrated reduced METH self-administration [36], reduced cocaine locomotor sensitization [69], and reduced the maintenance of METH CPP [70] following MTEP treatment. It appears mGlu5 is neither sufficient nor necessary for acquisition or maintenance of natural reward self-administration, as we found no effect of mGlu5 deletion on the acquisition and maintenance of sucrose self-administration. This is consistent with pharmacology - systemic MTEP does not reduce operant responding for a food reward [35]. Importantly, our study was designed to address the question of necessity. The data suggest that mGlu5 signalling is not necessary to acquire and/or support METH-driven behaviours. Nevertheless, we do not preclude the possibility that under other regimes/doses of METH an effect of genotype may emerge.

### mGlu5 Moderates Operant Extinction of METH but not Sucrose

Despite a lack of phenotype for operant self-administration of METH, mGlu5 KO mice displayed a clear deficit in the extinction of METH-seeking. The increased latency to extinguish the operant response for METH in mGlu5 KO mice suggests deficits in METH operant extinction learning, which essentially requires the animal to actively inhibit responding to a lever that was previously rewarding. Notably, this effect was not observed for sucrose, suggesting a differential role for mGlu5 in extinction learning for drug compared to natural rewards. This is important when considering mGlu5 as a potential therapeutic target, as it suggests mGlu5 may modulate drug-specific cognitive processes, without affecting cognitive processes involved in natural reward processing. Our findings are consistent with the literature; in rats, the mGlu5 PAM CDPPB facilitates extinction of cocaine CPP [59] and operant cocaine self-administration [58].

It is possible the operant extinction deficits observed are due to modulatory effects of mGlu5 on contextual salience. Indeed, mGlu5 may be necessary for learning to inhibit context-drug associations. That is, mGlu5 KO mice may have showed persistent operant responding during extinction despite the absence of drug, because previous context-METH associations were too salient in these mice compared to WTs. Support for this is provided by the elevated conditioned hyperactivity observed in the CPP test session, where KO mice responded with greater locomotor activity than WTs to a drug-paired environment in the absence of drug availability. Similarly, mGlu5 KO mice displayed exaggerated conditioned locomotor activity upon return to a cocaine-paired context in the absence of the drug [68]. Also, mGlu5 on dopamine D_1_ expressing neurons have been implicated in the acquisition of the incentive value of a conditioned stimulus, suggesting a role for mGlu5 in assigning valence to reward related stimuli [71]. Identifying which facets of extinction learning mGlu5 may be modulating is important for treatment of human addicts; the efficacy of cue exposure therapy is lacking (for a meta-analysis, see [72]), thus reducing the salience of drug-associated contexts may provide a more effective treatment approach to reducing relapse. Future experiments will undoubtedly address the role of mGlu5 in mediating the salience of cues and contexts associated with drug availability.

In contrast to METH, we found that mGlu5 KO and WT littermates demonstrated comparable extinction for a sucrose reinforcer. Our findings parallel those of Eiler et al. [73], where mGlu5 KO mice showed no differences in extinction of the operant response for sucrose pellets. The different phenotypes in mGlu5 KO mice in relation to extinction of operant responding for food and METH is presumably due to different neural adaptations which occur following self-administration and/or extinction of a drug versus a natural reinforcer. Self-administration of either cocaine or sucrose has the capacity to induce plasticity at glutamatergic synapses in midbrain dopamine neurons yet only in the case of cocaine does this potentiation persist beyond 21 days of abstinence [76]. Moreover, increased phosphorylation of the 2-amino-3-(3-hydroxy-5-methyl-isoxazol-4-yl)propanoic acid (AMPA) receptor subunit GluA1 (associated with the presence of high-conducting Ca^2+^ permeable GluA2-lacking AMPA receptors and thus changes in synaptic plasticity) is observed in other mesocorticolimbic regions (dorsal and ventral striatum) during cocaine, but not sucrose withdrawal [74]. Furthermore, firing rates in the NAcc are elevated following abstinence and during extinction from cocaine self-administration [75], but not sucrose [76]. It is possible that similar adaptations may occur following METH self-administration and/or extinction of METH self-administration. Deletion of mGlu5 may affect normal neural adaptations which occur in response to METH and/or operant extinction of METH, but not sucrose.

### Deletion of mGlu5 Enhances Propensity to Reinstate Operant METH- but not Sucrose-seeking

Upon exposure to cues signalling drug-availability, mGlu5 KO mice demonstrated a higher propensity to reinstate drug-seeking. mGlu5 KO mice demonstrated lower reinstatement magnitude (i.e. decreased active lever pressing); however, the low number of reinstating WT mice and the variability in their reinstatement magnitude complicates interpretation. While acute antagonism of mGlu5 signalling reduces the magnitude of reinstatement for METH [77], cocaine [78], ethanol [79] and nicotine [80], differences in the propensity to reinstate are rarely examined. Furthermore, numerous discrepancies between genetic and pharmacological studies using mGlu5 antagonists and mGlu5 KO mice have been documented (e.g. [41], [42]). A number of factors may make comparisons between acute pharmacological and genetic studies difficult; notably, tolerance to mGlu5 antagonists [61], [81]–[83] and receptor desensitization [82], [83] have been reported. Furthermore, it is possible that developmental compensation resulting from embryonic deletion of mGlu5 may affect the behaviours observed; mGlu5 KO mice show increased dendritic spine density [84], and increased spine diameter has been linked to cue-induced reinstatement [85].

The expression of extinction - and thus reinstatement propensity - likely depends upon contextual associations. Chaudhri and colleagues [86] demonstrated that if extinction occurs in multiple contexts (e.g. A, B, C), reinstatement is lower in a new context (D) than if extinction was only learnt in one context (A) for an equivalent period. Furthermore, Torregrossa *et al.* [87] demonstrated treatment with the NMDA agonist D-cycloserine enhances extinction in a different context, an effect mediated by the NAcc. This suggests glutamatergic tone in the accumbens, where mGlu5 is expressed, is important for the generalisation of extinction learning in one context to another. While our study did not examine extinction in multiple contexts, it is possible that mGlu5 KO mice failed to generalise what was learnt in the extinction context, where there was no discriminative cue and no CS, to the reinstatement context, where the discriminative cue and CS were present.

Furthermore, if there is a lack of generalisation in KO mice, this may be due to the discriminative cue, CS and operant chamber forming a compound stimulus representing drug availability (see [88]). During extinction, the repeated exposure to the context without METH served as context extinction sessions. However, the context in combination with the discriminative cue and CS (i.e., the reinstatement context) was never extinguished. The latter may present a compound cue signalling drug availability and hence the salience of the context. As discussed earlier, mGlu5 can regulate drug contextual salience [71]. If mGlu5 regulates or inhibits responding to drug-associated cues or contexts, then loss of mGlu5 signalling may result in a disproportionate significance given to these cues or contexts. This may explain why reinstatement occurred more reliably in KO compared to WT mice.

Intriguingly, WT mice demonstrated a lower propensity to reinstate to METH, but not sucrose, compared to mGlu5 KO mice. It is possible that in WT mice sucrose is a more preferable and potentially more salient reward compared to METH. Rats demonstrate a preference for saccharin self-administration over cocaine [89], [90]. This effect may be dependent on the training period because Galuska and colleagues [91] demonstrated METH self-administration and reinstatement of METH-seeking are enhanced by extended exposure, but not a shorter exposure period; an effect not present for sucrose (see also [92]). In that study, the demand curve (i.e. how self-administration decreases with increases in response requirements) for sucrose was higher than that of METH before extended exposure to both reinforcers, suggesting that a shorter period of exposure may make sucrose a more desired reinforcer than METH [91]. Hence, it is possible the self-administration period in the current study was not long enough to reliably induce reinstatement to METH in all WT mice, while at the same time being sufficient for robust reinstatement of sucrose-seeking. This is an important point; the sucrose reinstatement data provide validation of the paradigm in WT mice. Accordingly, the relatively modest reinstatement of METH-seeking in WT mice presumably reflects a comparatively lesser “value” than sucrose under the regime tested.

In comparison to METH, mGlu5 deletion had no effect on the propensity or magnitude of cue-induced reinstatement of sucrose-seeking. This is in accordance with a number of pharmacological studies [36], [78], [80], [93]; yet our findings did not replicate those of Eiler and colleagues [73], who demonstrated reduced reinstatement of food-seeking in mGlu5 KO mice. It is important to note that in the current study, if reinstatement criteria were not applied, mGlu5 KO mice demonstrated reduced cue-induced reinstatement for sucrose (data not shown). Reinstatement criteria were applied in the current study to provide a parallel for the METH self-administration findings and to accurately reflect the behavioural spectrum observed when analyzing individual mice compared to populations [94]. Also, procedural differences between the two studies may account for the divergence in phenotype. Importantly, the Eiler *et al.* study administered food rewards during the cue-induced reinstatement test, making the distinction between cue-induced and food-primed reinstatement unclear. The compound effect of food-primes and cue presentation may affect reinstatement in a different manner to each of these stimuli alone, and makes direct comparisons between the two studies difficult. Indeed, the paradigm used by Eiler and colleagues is essentially a study of reacquisition followed by rapid re-extinction more so than reinstatement.

### Possible Circuitry Modulating Extinction and Reinstatement Behaviour in mGlu5 KO Mice

mGlu5 signalling in a number of regions may be required to mediate extinction and reinstatement of operant METH-seeking. Extinction circuitry overlaps considerably with reinstatement circuitry [95]. This circuit connects the medial prefrontal cortex (infralimbic and prelimbic cortices) and basolateral amygdala to the NAcc core and shell, which projects to areas associated with motor output, the substantia nigra and ventral pallidum [95]–[99]. mGlu5 is expressed in most of the regions implicated in this circuit [31], [79], [100], [101]. Within this circuit, mGlu5 activity in the infralimbic (IL) and NAcc may be mediating the observed extinction and reinstatement phenotypes. Administration of the mGlu5 antagonist MPEP into the IL reduces recall of extinction learning [101]. The IL is recruited only after more than one day of extinction training [98]; the current study demonstrated a difference in extinction learning after day 1 (Fig. 3a), consistent with the notion that mGlu5 signalling in the IL modulates extinction. A reduction in mGlu5 activity in the IL may also be involved in the reinstatement phenotype observed; inactivation of IL promotes cue-induced reinstatement [98], while enhancement of AMPA activity in the IL suppresses cue-induced cocaine reinstatement [102].

Reduced mGlu5 signalling in the NAcc may also account for the extinction deficit and reinstatement propensity in mGlu5 KO mice. Suto and colleagues [103] demonstrated that extinction training enhances extracellular glutamate levels in the NAcc core and shell, compared to yoked saline and cocaine controls. Also, the extinction of cocaine self-administration results in reduced cell surface expression of mGlu5 in the NAcc core [104]. However, in contrast to our findings, pharmacological studies suggest reduced mGlu5 signalling in the NAcc reduces reinstatement, where we found the opposite effect. Intra-NAcc shell microinjections of MPEP reduce drug-primed reinstatement for cocaine [105], while mGlu5 agonist administration potentiates cue-induced reinstatement for cocaine [106]. Considering the critical role the NAcc core and shell play in reinstatement [10], the nature of the role of mGlu5 in the accumbens on reinstatement behaviour requires further investigation.

### Conclusion

The present study highlights a role for mGlu5 in the extinction and reinstatement of operant METH-, but not sucrose-, seeking. This, in combination with the enhanced conditioned hyperactivity during the CPP test, implicates mGlu5 in the contextual salience of drug-related cues and environments. Future studies will delineate the anatomic loci where mGlu5 signalling contributes to the extinction and reinstatement of METH-seeking.
